# Modern Cardiac Surgical Outcomes in Nonagenarians: A Multicentre Retrospective Observational Study

**DOI:** 10.3389/fcvm.2022.865008

**Published:** 2022-07-14

**Authors:** Laurence Weinberg, Dominic Walpole, Dong Kyu Lee, Michael D'Silva, Jian Wen Chan, Lachlan Fraser Miles, Bradly Carp, Adam Wells, Tuck Seng Ngun, Siven Seevanayagam, George Matalanis, Ziauddin Ansari, Rinaldo Bellomo, Michael Yii

**Affiliations:** ^1^Department of Anaesthesia, Austin Health, Melbourne, VIC, Australia; ^2^Department of Critical Care, The University of Melbourne, Melbourne, VIC, Australia; ^3^Department of Anesthesiology and Pain Medicine, Dongguk University Ilsan Hospital, Goyang, South Korea; ^4^Department of Cardiac Surgery, Epworth Eastern Hospital, Melbourne, VIC, Australia; ^5^Department of Cardiac Surgery, Austin Health, Melbourne, VIC, Australia; ^6^Department of Intensive Care, Epworth Eastern Hospital, Melbourne, VIC, Australia; ^7^Department of Intensive Care, Austin Health, Melbourne, VIC, Australia

**Keywords:** cardiac surgery, anesthesia, nonagenarians, outcomes, complications, mortality, acute kidney injury, delirium

## Abstract

**Background:**

There have been multiple recent advancements in the selection, optimisation and management of patients undergoing cardiac surgery. However, there is limited data regarding the outcomes in nonagenarians, despite this cohort being increasingly referred for these interventions. The objective of this study was to describe the patient characteristics, management and outcomes of a cohort of nonagenarians undergoing cardiac surgery receiving contemporary peri-operative care.

**Methods:**

After receiving ethics approval, we conducted a retrospective observational study of nonagenarians who had undergone cardiac surgery requiring a classic median sternotomy. All operative indications were included. We excluded patients who underwent transcatheter aortic valve implantation (TAVI), and surgery on the thoracic aorta *via* an endovascular approach (TEVAR). Patients undergoing TEVAR often have the procedure done under sedation and regional blocks with local anesthetic solution. There is no open incision and these patients do not require cardiopulmonary bypass. We also excluded patients undergoing minimally invasive mitral valve surgery *via* a videoscope assisted approach. These patients do not have a median sternotomy, have the procedure done *via* erector spinae block, and often are extubated on table. Data were collected from four hospitals in Victoria, Australia, over an 8-year period (January 2012–December 2019). The primary objective was to assess 6-month mortality in nonagenarian patients undergoing cardiac surgery and to provide a detailed overview of postoperative complications. We hypothesized that cardiac surgery in nonagenarian patients would be associated with a 6-month postoperative mortality <10%. As a secondary outcome, we hypothesized that significant postoperative complications (i.e., Clavien Dindo Grade IIIb or greater) would occur in > 30% of patients.

**Results:**

A total of 12,358 adult cardiac surgery patients underwent surgery during the study period, of whom 18 nonagenarians (0.15%) fulfilled inclusion criteria. The median (IQR) [min-max] age was 91.0 years (90.0:91.8) [90–94] and the median body mass index was 25.0 (kg/m^2^) (22.3:27.0). Comorbidities, polypharmacy, and frailty were common. The median predicted mortality as per EuroSCORE-II was 6.1% (4.1:14.5). There were no cases of intra-operative, in-hospital, or 6-month mortality. One (5.6%) patient experienced two Grade IIIa complications. Three (16.7%) patients experienced Grade IIIb complications. Three (16.7%) patients had an unplanned hospital readmission within 30 days of discharge. The median value for postoperative length of stay was 11.6 days (9.8:17.6). One patient was discharged home and all others were discharged to an inpatient rehabilitation facility.

**Conclusion:**

In this selected, contemporary cohort of nonagenarian patients undergoing cardiac surgery, postoperative 6-month mortality was zero. These findings support carefully selected nonagenarian patients being offered cardiac surgery (Trials Registry: https://www.anzctr.org.au/ACTRN12622000058774.aspx).

## Introduction

Very elderly patients undergoing cardiac surgery are vulnerable (1). However, as life expectancy continues to rise in resource rich countries, the global population of nonagenarians is expected to increase from 16.3 million in 2015 to an estimated 30.9 million in 2030. (2) Additionally, the proportion of the over-75 population being offered, and accepting surgical intervention is increasing (3, 4). Accordingly, the number of nonagenarians presenting for surgery is also expected to increase. These demographic changes present clinical challenges.

Advanced age increases the risks of surgery, with a linear association between advanced age and worse postoperative outcomes (1). Advanced age is associated with physiological derangements and a greater burden of comorbidity. The estimated postoperative survival rates after surgery for nonagenarians at one, 3–5 years are 59.6, 35.8 and 24.1%, respectively (5, 6). Such studies, however, have examined individuals undergoing non-cardiac surgery, (7, 8) and have not provided granular insights into the anesthetic and peri-operative course of cardiac surgical patients. This is a significant knowledge gap, and there is little evidence to guide and inform the patient selection and peri-operative care of nonagenarians undergoing cardiac surgery.

Accordingly, we conducted a retrospective observational study to describe the peri-operative care and postoperative outcomes of nonagenarians undergoing cardiac surgery.

## Materials and Methods

### Study Objectives

The primary objective of this study was to determine the 6-month mortality in nonagenarian patients undergoing cardiac surgery, and to provide a detailed overview of postoperative complications.

The secondary aims were to describe peri-operative management, including patient characteristics, comorbidity burden, indications for surgery, anesthesia medications, use of fluids, vasoactive drugs and inotropes, and postoperative course.

We hypothesized that cardiac surgery in nonagenarian patients would be associated with postoperative mortality within 6 months of surgery of <10%. Secondarily, we hypothesized that significant postoperative complications–defined as the development of a Clavien Dindo Grade IIIb or greater complications–would occur in >30% of patients.

### Data Sources and Processing

We conducted a multicentre retrospective study of nonagenarians who had undergone cardiac surgery requiring a classic median sternotomy. We excluded patients undergoing minimally invasive mitral valve or aortic surgery. These patients are younger, and this procedure undertaken *via* a thoracoscopic approach, often with a with erector spinae block. Accordingly, they do not have a midline sternotomy incision. We excluded patients undergoing surgery on the thoracic aorta *via* an endovascular approach (TEVAR) and patients undergoing transfemoral aortic valve replacements (TAVR). These patients have the procedure under sedation and regional blocks with local anesthetic solution. There is no open incision and these patients do not require cardiopulmonary bypass.

Data were collected from three hospitals in Melbourne, Victoria, Australia, over an 8-year period from 1 January 2012 to 31 December 2019. These hospitals were Austin Hospital, Warringal Hospital and Epworth Eastern Hospital. All hospitals are university teaching institutions, and all procedures were undertaken by a dedicated multidisciplinary team of consultant cardiac anesthetists, surgeons, and intensivists, who work across all three institutions. The study was approved by 1the Austin Health Human Research Ethics Committee (HREC 21/30). Written informed consent was waived as only de-identified data was collected. The study was registered with the Australian New Zealand Clinical Trials Registry (https://www.anzctr.org.au/ACTRN12622000058774.aspx). Data were collected from a prospectively managed, electronic surgical database (Cerner Millenium®, Missouri, USA) and from the scanned medical records of anesthesia charts. A team of two experienced clinicians reviewed and crosschecked the data. Two other clinicians checked each record independently.

We included patients' preoperative characteristics, peri-operative laboratory values, surgical data, and intensive care unit (ICU) admission data. Socio-economic status and frailty were also assessed and recorded. Risk factors, preoperative medications, and comorbidities relevant to suitability for surgery were recorded, and operative risk calculated. Key features of the preoperative paradigm, including results of recent blood testing, transthoracic echocardiography and coronary angiography, were collated if available. The time from admission to surgery was also recorded. An analysis of postoperative complications, in-hospital mortality and readmissions was performed. Discharge destination was obtained, as was any record of follow-up and the time from surgery to the last follow-up encounter.

### Definitions

‘Rural' residence was defined as Modified Monash Model Category 2 or higher (9). Socio-economic status was assessed based on the national percentile rank of patients' residential postcode in the Australian Bureau of Statistics Socio-economic Indexes for Areas Index of Relative Socio-economic Advantage and Disadvantage, with higher rankings indicating greater advantage and lesser disadvantage on specific socio-economic measures relative to other areas (10). Frailty was defined as a score of 5 or higher on the Canadian Study on Health and Aging Clinical Frailty Scale (11).

Previous myocardial infarction was defined as “recent” or “remote” if it occurred within or more than 90 days, respectively, of the index operation (12).

Heart failure was characterized using the Heart Foundation of Australia guidelines (13). Extracardiac arteriopathy was defined as one or more of the following: claudication; carotid occlusion or > 50% stenosis; previous amputation for arterial disease; and previous or planned intervention on abdominal aorta, limb arteries or carotids (12). Chronic kidney disease was defined and staged according to the glomerular filtration rate criteria in the Kidney Disease: Improving Global Outcomes (KDIGO) 2012 guideline for chronic kidney disease (14). Anemia was defined according to World Health Organization criteria (15). The overall burden of comorbidity was quantified using the Charlson Comorbidity Index (16).

The total number of medications included prescribed, over-the-counter and as-required medications administered by oral, inhaled, rectal, vaginal, intramuscular, subcutaneous or intravenous routes. Topical medications including creams, lotions and ointments for wound care and eye drops were not recorded. Polypharmacy was defined as five or more medications (17).

The urgency of surgery was considered ‘elective' for patients admitted routinely for the operation, ‘urgent' for those not electively admitted for the operation but requiring surgery during the current admission and ‘emergent' for those requiring surgery before the beginning of the next working day after the decision to operate. After-hours surgery was defined as surgery commencing or finishing between 1800 and 0800 h.

The American Society of Anesthesiologists (ASA) physical status classification for patients was based on the treating anesthetists' assessment immediately prior to surgery. “Critical preoperative state” denoted patients having one or more of the following in the same hospital admission as the operation: ventricular fibrillation, ventricular tachycardia or aborted sudden death; cardiac massage; ventilation before arrival to the operating room; inotropes; intra-aortic balloon pump or ventricular-assist device before arrival to the operating room; or acute renal failure (anuria or oliguria < 10 mL/hr) (12). The predicted operative risk of in-hospital mortality was calculated for each patient using the EuroSCORE II risk model (12). The estimated risk of requiring renal replacement therapy (RRT) postoperatively was calculated using the Cleveland Clinic Score (18).

A complication was defined as any deviation from the normal postoperative course and graded using the Clavien–Dindo classification system (19). “Return to theatre” was defined as surgery within 30 days of the index operation due to a related complication, and ‘readmission' was defined as an unplanned admission within 30 days of discharge. Acute kidney injury (AKI) was defined and staged according to the KDIGO 2012 guidelines for AKI (14).

Time to surgery was defined as the time from admission to the start of anesthesia. Duration of mechanical ventilation was defined as the time from the end of surgery to tracheal extubation. Time to first oral intake and time to first mobilization to chair were measured from the end of surgery. The postoperative and total lengths of stay were defined as the time from surgery to discharge and the time from admission to discharge, respectively.

### Statistics

Statistical analysis was performed using IBM SPSS Statistics for Windows, version 23 (IBM Corp., 2015, Armonk, NY, USA) and Stata/SE 13.0 for Windows (StataCorp LP, Texas, USA, 2013). Data were de-identified, variable names were encrypted, and all data were coded with numerical values to blind the collected variables' characteristics to the statistician. The descriptive statistics presented below are shown as either median (with interquartile range; minimum–maximum in parentheses) or number (and percentage in parentheses). Only non-zero data points were included in the calculation of descriptive statistics for numerical variables (e.g., drug doses and periods). The study is reported following The Strengthening the Reporting of Observational Studies in Epidemiology (STROBE) guidelines (20).

## Results

### Preoperative Paradigm

Over the study period 12,391 adult patients were considered for open cardiac surgery *via* a median sternotomy approach, of whom 49 (0.39%) were nonagenarians. There were no nonagenarians who underwent minimally invasive aortic or mitral surgery, or TEVAR. Thirty-three patients were considered for TAVR, of which 27 underwent this procedure for severe symptomatic aortic stenosis. Six patients with severe aortic stenosis were deemed at prohibitive risk for both SAVR and TAVR and were accordingly managed conservatively. Of the remaining 2,358 patients who underwent open cardiac surgery, 18 (0.15%) nonagenarians fulfilled the inclusion criteria. Two nonagenarians underwent isolated surgical aortic valve replacement (SAVR). Both these patients had critical symptomatic aortic stenosis and transfemoral access, valve and aortic morphology were unfavorable for TAVR. Of the remaining patients who underwent open cardiac surgery, 12 patients underwent coronary artery bypass grafting (CABG, of which four had isolated CABG, two had CABG plus left atrial appendage ligation and or a MAZE procedure, and six patients underwent CABG plus single or double valve replacement. One patient had a double valve replacement, and one patient had a triple valve replacement.

The demographics, socio-economic status and comorbidities of the 18 patients who underwent open cardiac surgery are summarized in Table 1. The median (IQR) [min-max] age was 91 years (90.0:91.8) [90–94] and the median (IQR) body mass index was 25.0 kg/m^2^ (22.3:27.0). Most were men. Most patients resided in areas ranked above the 50th socioeconomic percentile relative to the rest of the nation. Approximately half were frail. Baseline characteristics of nonagenarians who underwent TAVR in our institution are also present in Table 1.

**Table 1:** Demographics and comorbidities of nonagenarians undergoing open cardiac surgery *via* a sternotomy and transcatheter aortic valve replacement.

**Variable**	**Open cardiac surgery**	**Transcatheter aortic valve**
	**(*N* = 18)**	**replacement (*N* = 27)**
**Surgical procedure**		
Aortic valve replacement (isolated)	2 (11%)	27 (100%)
Aortic valve replacement, ascending aorta replacement	2 (11%)	N/A
Aortic valve and mitral replacement, tricuspid valve repair, left atrial Maze procedure, ligation of left atrial appendage	1 (6%)	N/A
Aortic valve replacement, CABG	2 (11%)	N/A
Aortic valve replacement, CABG, ligation of left atrial appendage	2 (11%)	N/A
Aortic valve replacement, CABG, tricuspid valve repair, left ventricular epicardial pacing lead	1 (6%)	N/A
CABG (isolated)	4 (22%)	N/A
CABG, ligation left atrial appendage	1 (6%)	N/A
CABG, ligation left atrial appendage, left atrial Maze procedure	1 (6%)	N/A
CABG, mitral valve replacement, ligation of left atrial appendage, closure of patent foramen ovale	1 (6%)	N/A
Mitral valve replacement, tricuspid valve repair, ligation of left atrial appendage, closure of patent foramen ovale	1 (6%)	N/A
**Patient characteristics**		
Age	91.0 (90.0:91.8), [90–94)	92.0 (90.0:93); [90–98]
Female	4 (22%)	11 (40.7%)
Male	14 (78%)	16 (59.3%)
Body mass index (kg/m^2^)	25.0 (22.3:27.0), [18–30]	24.0 (22.1:25.0); [17–31]
**Residential status**		
Home	17 (94%)	26 (96.3%)
Residential care facility	1 (6%)	1 (0.7%)
**Rurality and socio-economic status**		
Rural residence	6 (33%)	4 (9.4%)
MMM category	1.0 (1.0:2.0), [1–5]	1.0 (1.0:2.0), [1–4]
SEIFA (IRSAD) percentile	78 (56:89), [21–94]	77 (55:88), [27–93]
**Frailty**		
Mild or greater	10 (56%)	7 (25.6%)
CSHA frailty grade	5.0 (4.0:5.0), [4–6]	3.0 (2.0:4.0), [3–4]
**Comorbidities**		
Hypertension	12 (67%)	13 (48.1%)
Atrial fibrillation	5 (28%)	1 (3.7%)
Recent myocardial infarction	4 (22%)	0
History of myocardial infarction	2 (11%)	2 (7.4%)
Previous PCI	5 (28%)	1 (4.2%)
Previous cardiac surgery	0 (0%)	0
Permanent pacemaker	3 (17%)	1 (3.7%)
Heart failure	12 (67%)	4 (29.6%)
**NYHA functional class**		
1	0 (0%)	5 (20.9%)
2	6 (33%)	13 (18.5%)
3	11 (61%)	6 (22.2%)
4	1 (6%)	0
Stroke/TIA	2 (11%)	2 (7.4%)
Current smoker	0 (0%)	0
Past smoker	7 (39%)	4 (29.6%)
Extracardiac arteriopathy	3 (17%)	0
Chronic lung disease	4 (22%)	0
Diabetes mellitus	4 (22%)	2 (7.4%)
Chronic kidney disease	11 (61%)	8 (29.6%)
**Chronic kidney disease (stage)**		
1	0 (0%)	5 (18.5%)
2	0 (0%)	3 (11.2%)
3a	4 (22%)	0
3b	5 (28%)	0
4	2 (11%)	0
5	0 (0%)	0
Anemia	11 (61%)	9 (33.3%)
Neurological/musculoskeletal dysfunction	3 (17%)	0
Chronic liver disease	0 (0%)	0
Dementia	1 (6%)	0
Localised solid malignancy	2 (11%)	0
Leukemia	2 (11%)	0
**Charlson comorbidity Index**		
Score	6.0 (5.0:6.8), [4–9]	6.0 (5.0:6.2), [5–10]

*Binary variables are presented as n (%) patients, and numerical variables are presented as Mdn [interquartile range], range (minimum–maximum). MMM, modified monash model; SEIFA (IRSAD), socio-economic indexes for areas (index of relative socio-economic advantage and disadvantage); CSHA, Canadian study of health and ageing; PCI, percutaneous coronary intervention; NYHA, New York heart association; TIA, transient ischemic attack; CCI, charlson comorbidity index*.

In nonagenarians undergoing open cardiac surgery, hypertension, heart failure, chronic kidney disease and anaemia were the most common comorbidities. Previous myocardial infarction and percutaneous coronary intervention were also frequent. Only one patient had a diagnosis of mild dementia. A multidisciplinary team comprising a cardiac surgeon, cardiologist, anaesthetist and geriatrician assessed all patients preoperatively and sought to optimise their preoperative condition.

The participants' preoperative medications are summarised in Table 2. Polypharmacy was common. Preoperative hematological and biochemical results, echocardiography and coronary angiography findings are summarised in Table 3. The median haemoglobin was slightly below normal. The baseline estimated glomerular filtration rate was <60 mL/min/1.73 m^2^ for more than half of patients, with two (11%) patients recording values <30mL/min/1.73 m^2^. Significant valve disease was reported in 11 (61%) patients, predominantly severe aortic stenosis, followed by severe mitral regurgitation. Regional wall motion abnormalities were recorded in one-third of patients.

**Table 2:** Preoperative medications of nonagenarians undergoing open cardiac surgery *via* a sternotomy.

**Medication**	**Open cardiac surgery**
	**(*N* = 18)**
Aspirin	13 (72%)
Beta blocker	11 (61%)
ACEi/ARB	8 (44%)
Statin	8 (44%)
Nitrites/antianginals	7 (39%)
Frusemide	7 (39%)
Dihydropyridine CCB	6 (33%)
P2Y12 inhibitor (clopidogrel)	6 (33%)
Proton pump inhibitor	5 (28%)
Inhaled bronchodilators or steroids	4 (22%)
Other antiarrhythmic	3 (17%)
Oral diabetes medications	3 (17%)
Direct-acting oral anticoagulant	2 (11%)
Insulin	2 (11%)
Fibrate	1 (6%)
Warfarin	1 (6%)
Polypharmacy	13 (72%)
Total number of medications	6.5 (4.3:8.8), [0–16]

*Binary variables are presented as n (%) patients, and numerical variables are presented as Mdn [interquartile range], range (minimum–maximum). ACEi, angiotensin-converting enzyme inhibitor; ARB, angiotensin receptor blocker; CCB, calcium channel blocker*.

**Table 3:** Preoperative variables of nonagenarians undergoing open cardiac surgery.

**Variable**	**Open cardiac surgery (*N* = 18)**
**Preoperative blood testing**	
Hemoglobin (g/L)	123 (114:133), [98–140]
Platelets	235 (206:292), [165–372]
Albumin (g/L)	36 (34:37), [29–40]
International normalised ratio	1.1 (1.1:1.2), [1.0–1.4]
Ferritin (mmol/L)	109 (69:150), [41–219]
Troponin (mmol/L)	853 (43:896), [15–2,445]
B-type natriuretic peptide (pg/mL)	126 (111:137), [27–499]
**Estimated glomerular filtration rate (mL/min/1.73 m** ^**2**^ **)**	
≥90	1 (6%)
60–89	7 (39%)
45–59	4 (22%)
30–44	4 (22%)
15–29	2 (11%)
<15	0 (0%)
**Preoperative transthoracic echocardiography**	
Transthoracic echocardiogram available	16 (89%)
Left ventricular ejection fraction (%)	
>50	9 (56%)
1–50	5 (22%)
21–30	1 (6%)
≤ 20	1 (6%)
Left ventricular hypertrophy	10 (56%)
Left ventricle dilatation	1 (6%)
Left atrium dilatation	14 (78%)
Right ventricle systolic dysfunction	2 (11%)
**Estimated pulmonary artery pressure (mmHg)**	
≤ 30	7 (39%)
31–54	8 (44%)
≥55	1 (6%)
**Valve function**	
Significant valve disease	11 (61%)
**Aortic stenosis**	
Mild	2 (11%)
Moderate	1 (6%)
Severe	8 (44%)
**Aortic regurgitation**	
Mild	4 (22%)
Moderate	4 (22%)
Severe	0 (0%)
**Mitral regurgitation**	
Mild	6 (33%)
Moderate	1 (6%)
Severe	3 (17%)
**Tricuspid regurgitation**	
Mild	5 (28%)
Moderate	3 (17%)
Severe	1 (6%)
Regional wall motion abnormality	6 (33%)
**Preoperative coronary angiography**	
Coronary angiogram available	14 (78%)
Coronary vascular disease	11 (61%)
Significant coronary stenosis	10 (56%)
**Locations of significant stenoses**	
Left main	3 (17%)
Left anterior descending artery	9 (50%)
Left circumflex artery	6 (33%)
Right coronary artery	8 (44%)
Obtuse marginal	3 (17%)
First diagonal	4 (22%)
Posterior descending artery	1 (6%)
**Extent of coronary disease**	
Severe single vessel disease	0 (0%)
Severe double vessel disease	2 (11%)
Severe triple vessel disease	7 (39%)
Left ventricular end diastolic pressure ≥ 15 mmHg	3 (17%)
**Preoperative length of stay**	
Time from admission to surgery (days)	1.0 (0.7:6.4), [0.1–19.9]

*Binary variables are presented as n (%) patients, and numerical variables are presented as Mdn [interquartile range], range (minimum–maximum)*.

### Preoperative Operative Risk

Details of the type of surgery performed, participants' preoperative status and predicted operative risk are presented in Table 4. Approximately half of the surgeries were elective, one-third were urgent, and two were emergent. Almost half of surgeries occurred out of hours. Preoperatively, almost all patients were assigned an ASA physical status class of 4. One patient was in a critical preoperative state, requiring mechanical ventilation and a noradrenaline infusion during transportation to the operating theatre. The median EuroSCORE II predicted in-hospital mortality was 6.1% (4.1:14.5) [1.8–34.3].

**Table 4:** Preoperative status and operative risk of nonagenarians undergoing open cardiac surgery.

**Variable**	**Open cardiac surgery (*N* = 18)**
**Urgency of surgery**	
Elective	10 (56%)
Urgent	6 (33%)
Emergent	2 (11%)
**Timing of surgery**	
Out-of-hours surgery	8 (44%)
**Preoperative status**	
ASA class	
2	1 (6%)
3	3 (17%)
4	13 (72%)
5	1 (6%)
Critical preoperative state	1 (6%)
**Operative risk**	
EuroSCORE II predicted in-hospital mortality (%)	6.1 (4.1:14.5), [1.8–34.3]
Cleveland Score risk of dialysis (%)	1.8 (0.8:7.8), [0.4–7.8]

*Binary variables are presented as n (%) patients, and numerical variables are presented as Mdn [interquartile range], range (minimum–maximum). CABG, coronary artery bypass grafting; ASA, American society of anesthesiologists; EuroSCORE II, European system for cardiac operative risk evaluation II*.

### Intraoperative and Postoperative Management

Key features of postoperative management are summarised in Tables 5, 6. All patients had an arterial line inserted prior to induction of anesthesia. Vascular access for all patients included a pulmonary artery catheter, central venous catheter, and a large-gauge peripheral cannula. Vascular access lines were inserted prior to induction in more than half of patients. Prophylactic antibiotics were administered to all patients prior to skin incision. All operations were conducted on cardiopulmonary bypass with cardioplegia administered and patients heparinised then reversed with protamine. Transoesophageal echocardiography was performed immediately before and after cardiopulmonary bypass in all patients. Cardiac output was monitored intraoperatively by thermodilution for all patients.

**Table 5:** Intraoperative variable of nonagenarians undergoing open cardiac surgery.

**Variable**		**Open cardiac surgery (*N* = 18)**
**Duration**		
Total surgery time (minutes)		270 (229:341), [180–450]
Cardiopulmonary bypass time (minutes)		121 (95:158), [57–251]
Cross-clamp time (minutes)		95 (74:143), [48–231]
**Monitoring**		
Lowest pH on bypass		7.33 (7.30:7.38), [7.27–7.47]
Lowest temperature on bypass (°C)		33.5 (33.4:34.0), [28.1–34.5]
**Anaesthesia**		
Premedication		17 (94%)
Induction agent		
Fentanyl		12 (67%)
Propofol		8 (11%)
Alfentanil		4 (22%)
Remifentanil		2 (11%)
Ketamine		1 (6%)
Volatile anesthesia only		
Sevoflurane		10 (56%)
Intravenous anesthesia only		
Propofol		1 (6%)
Remifentanil		1 (6%)
Remifentanil + propofol + ketamine		1 (6%)
Volatile and intravenous anaesthesia combined		
Propofol + sevoflurane		4 (22%)
Propofol + isoflurane		1 (6%)
Muscle relaxant		
Rocuronium	No. of patients (%)	14 (78%)
	Total dose (mg)	100 (100:100), [50–150]
Pancuronium	No. of patients (%)	2 (11%)
	Total dose (mg)	8.0, 12.0
Cisactracurium	No. of patients (%)	2 (11%)
	Total dose (mg)	10.0, 40.0
**Vasoactive medications**		
Any vasoactive		15 (83%)
Metaraminol	No. of patients (%)	8 (44%)
	Total dose (mg)	1.0 (0.5:1.1), [0.5–3.0]
Noradrenaline	No. of patients (%)	5 (28%)
	Total dose (mg)	0.1 (0.1:1.2), [0.1–2.1]
Milrinone	No. of patients (%)	3 (17%)
	Total dose (mg)	2.0, 2.7, 3.0
Ephedrine	No. of patients (%)	2 (11%)
	Total dose (mg)	9.0, 24.0
Adrenaline	No. of patients (%)	1 (6%)
	Total dose (mcg)	45.0
Atropine	No. of patients (%)	1 (6%)
	Total dose (mg)	0.3
**Opioids**		
Any opioid		18 (100%)
Intravenous morphine equianalgesic dose (mg)		33 (28:54), [0.1–83]
Morphine	No. of patients (%)	6 (33%)
	Total dose (mg)	10 (10:10), [5–10]
Fentanyl	No. of patients (%)	12 (67%)
	Total dose (mcg)	625 (500:1,000), [350–1,250]
Alfentanil	No. of patients (%)	4 (22%)
	Total dose (mg)	20 (17:20), [7–20]
Remifentanil	No. of patients (%)	2 (11%)
	Total dose (mcg)	Not applicable
**Intravenous fluids**		
Any intravenous fluids		14 (78%)
Crystalloids (CSL)	No. of patients (%)	10 (56%)
	Total volume (L)	1.0 (1.0:1.7), [0.3–3.3]
Colloids (4% albumen)	No. of patients (%)	10 (56%)
	Total volume (L)	0.5 (0.5:0.5), [0.2–0.5]
**Blood products**		
Unprocessed residual pump blood		
No. of patients (%)	18 (100%)	
Total volume (mL)	566 (467:677), [400–720]	
Any blood products		11 (61%)
Red blood cells	No. of patients (%)	8 (44%)
	No. of units	2.0 (1.8:2.3), [1–7]
Platelets	No. of patients (%)	6 (33%)
	No. of units	2.0 (1.3:2.0), [1–3]
Cryoprecipitate	No. of patients (%)	5 (28%)
	No. of units	5.0 (5.0:6.0), [5–10]
Fresh frozen plasma	No. of patients (%)	2 (11%)
	No. of units	2, 3
Prothrombinex	No. of patients (%)	5 (28%)
	Total dose (units)	1,500 (1,000:2,000), [1,000–3,000]
**Other therapies**		
Antifibrinolytic		17 (94%)
Insulin		3 (17%)
Diuretic		4 (22%)
Desmopressin		1 (6%)

*Binary variables are presented as n (%) patients. Except where noted, numerical variables are presented as Mdn [interquartile range], range (minimum–maximum) and only include non-zero results. Where there are fewer than four data points for a numerical variable, individual patient results are listed. CSL, compound sodium lactate*.

**Table 6:** Postoperative management of nonagenarians undergoing open cardiac surgery.

**Variable**		**Open cardiac surgery (*N* = 18)**
**Recovery parameters**		
Duration of mechanical ventilation (hours)		10.0 (8.1:13.2), [3.5–33.3]
Time to first oral intake (hours)		15.4 (14.5:20.9), [9.2–40.8]
Time to first mobilize to chair (hours)		27.0 (18.1:41.5), [13.5–61.5]
Time to removal of drain tubes (days)		1.9 (1.7:2.1), [1.0–4.7]
Duration of epicardial pacing time (days)		1.7 (0.5:3.3), [0.3–6.2]
Duration of inotrope or vasopressor support (days)		2.1 (1.8:3.5), [0.4–6.7]
Length of stay in intensive care unit (days)		2.6 (2.1:4.6), [0.9–7.0]
**Other parameters**		
Lowest hemoglobin (g/L)		80 (79:84), [73–109]
Lowest temperature/Temp on arrival (°C)		35.0 (34.5:35.1), [33.2–35.4]
Lowest pH		7.31 (7.25:7.33), [7.22–7.36]
Highest lactate (mmol/L)		2.7 (1.8:3.0), [1.7–5.0]
**Vasoactive medications**		
Any vasoactive drug		14 (78%)
Noradrenaline	No. of patients (%)	12 (67%)
	Total dose (mg)	12.1 (4.7:31.0), [0.4–67.0]
Milrinone	No. of patients (%)	5 (28%)
	Total dose (mg)	18.1 (12.2:29.6), [5.8–124.5]
Metaraminol	No. of patients (%)	2 (11%)
	Total dose (mg)	0.5, 0.5
Glyceryl trinitrate	No. of patients (%)	1 (6%)
	Total dose (mg)	257.5
Nitroprusside	No. of patients (%)	1 (6%)
	Total dose (mg)	39.0
**Opioids**		
Any opioid		18 (100%)
Oral morphine equianalgesic dose (mg)		103 (67:251), [8–580]
Morphine (IV)	No. of patients (%)	8 (44%)
	Total dose (mg)	6.0 (3.3:11.3), [1–30]
Oxycodone (PO)	No. of patients (%)	14 (78%)
	Total dose (mg)	33 (10.6:102), [5–360]
Tapentadol	No. of patients (%)	1 (6%)
	Total dose (mg)	1,000
Tramadol (IV/PO)	No. of patients (%)	8 (44%)
	Total dose (mg)	300 (188:500), [100–500]
Fentanyl	No. of patients (%)	3 (17%)
	Total dose (mcg)	120, 140, 180
**Intravenous fluids**		
Any intravenous fluids		18 (100%)
Crystalloids (CSL)	No. of patients (%)	4 (22%)
	Volume (L)	1.0 (0.8:1.0), [0.3–1.2]
Colloids (4% albumin)	No. of patients (%)	16 (89%)
	Volume (L)	1.4 (1.0:1.5), [0.5–2.0]
**Blood products**		
Red blood cells	No. of patients (%)	14 (78%)
	No. of units	2.0 (1.3:3.0), [1.0–7.0]
Platelets	No. of patients (%)	2 (11%)
	No. of units	1, 1
Cryoprecipitate	No. of patients (%)	1 (6%)
	No. of units	3
Fresh frozen plasma	No. of patients (%)	1 (6%)
	No. of units	2
**Other interventions**		
Tracheostomy		0 (0%)
Reintubation		0 (0%)
Non-invasive ventilation		2 (11%)
High-flow nasal cannula		3 (17%)
Antibiotics		18 (100%)
Antiarrhythmics		13 (72%)
Diuretic		13 (72%)
Vasopressin		1 (6%)
Anticoagulation		1 (6%)
Intra-aortic balloon pump		0 (0%)
Renal replacement therapy		0 (0%)

*Binary variables are presented as n (%) patients. Except where noted, numerical variables are presented as Mdn [interquartile range], range (minimum–maximum) and only include non-zero results. IV, intravenous; PO, per os (oral administration); CSL, compound sodium lactate*.

### Complications, Inpatient Mortality, Length of Stay and Follow-Up

A summary of postoperative complications, lengths of stay, discharge destinations and follow-up are provided in Table 7. For nonagenarians undergoing open cardiac surgery, all patients except one suffered a postoperative complication. The most common complication was AKI (Stage 1 in five patients, Stage 2 in six patients and Stage 3 in two patients). New atrial fibrillation and/or flutter and delirium were also common complications. No patients required reintubation, tracheostomy, intra-aortic balloon counterpulsation or renal replacement therapy in the postoperative period. Postoperatively, most patients were treated with diuretics (typically furosemide) and antiarrhythmics (generally amiodarone and/or metoprolol).

**Table 7:** Complications, inpatient mortality, length of stay and follow-up of nonagenarians undergoing open cardiac surgery and transcatheter aortic valve replacement.

**Variable**	**Open cardiac surgery**	**Transcatheter aortic valve replacement**
	**(*N* = 18)**	**(*N* = 27)**
**Complications**		
Any complication	17 (94%)	15 (55.5%)
Number of complications	2.0 (2.0:3.0), [1–4]	1.0 (0.0:2.0), [0–5]
Return to theatre	3 (17%)	1 (3.7%)
Readmission	3 (17%)	1 (3.7%)
**Complications by type**		
Vascular access injury requiring intervention	0 (0%)	2 (7.4%)
Acute kidney injury	13 (72%)	5 (18.5%)
New atrial fibrillation and/or atrial flutter	8 (44%)	1 (3.7%)
Delirium	7 (39%)	1 (3.7%)
Pneumonia	3 (17%)	1 (3.7%)
Heart block and/or bradycardia requiring pacemaker	2 (11%)	1 (3.7%)
Pleural effusion	1 (6%)	0 (0%)
Gastrointestinal haemorrhage	1 (6%)	0 (0%)
Pneumothorax	1 (6%)	0 (0%)
Wound infection	1 (6%)	2 (7.4%)
Wound hematoma	1 (6%)	2 (7.4%)
Pressure injury	1 (6%)	0 (0%)
Urinary tract infection	1 (6%)	0 (0%)
**Complications by severity (Clavien–Dindo classification)**		
I	14 (78%)	3 (11.1%)
II	10 (56%)	10 (37.0%)
IIIa	1 (6%)	1 (3.7%)
IIIb	3 (17%)	1 (3.7%)
IV	0 (0%)	0 (0%)
V	0 (0%)	0 (0%)
**Acute kidney injury by severity (KDIGO criteria)**		
Stage 1	5 (28%)	3 (11.1%)
Stage 2	6 (33%)	2 (7.4%)
Stage 3	2 (11%)	0 (0%)
**Inpatient mortality**		
Intraoperative	0 (0%)	0 (0%)
In-hospital	0 (0%)	0 (0%)
**Length of stay**		
Postoperative (days)	11.6 (9.8:17.6), [6.8–23.0]	3.1 (2.9:4.9), [1.1–28.3]
**Discharge destination**		
Home	1 (6%)	25 (92.6%)
Inpatient rehabilitation facility	17 (94%)	2 (7.4%)
**Follow-up**		
Mortality at 6 months	0 (0%)	1 (3.7%)
Record of follow-up > 6 months after surgery	10 (55%)	27 (100%)
Time to recorded last follow-up (months)	11.3 (6.0:26.4), [6.0–69.4)	29.2 (18.2:39.5), [12–46]

*Binary variables are presented as n (%) patients. Numerical variables are presented as Mdn [interquartile range], range (minimum–maximum) and only include non-zero results. KDIGO, kidney disease: improving global outcomes*.

Overall, 14 (78%) patients suffered at least one Grade I complication, and 10 (56%) patients suffered at least one Grade II complication. One (6%) patient experienced two Grade IIIa complications: pneumothorax and pleural effusion—both requiring separate radiologically guided insertion of an intercostal catheter. Three (17%) patients experienced Grade IIIb complications: two underwent insertion of a permanent pacemaker, and one underwent gastroscopy and hemostatic clipping for gastric haemorrhage. There were no cases of intraoperative or in-hospital mortality.

Three (17%) patients had an unplanned hospital readmission within 30 days of discharge: one with AKI and *Enterococcus faecalis* bacteraemia secondary to a urinary tract infection, one with an infective exacerbation of chronic obstructive pulmonary disease and one with shingles and a pleural effusion. All three patients had an additional diagnosis of congestive cardiac failure.

The median values for the postoperative length of stay and total length of stay were 11.6 and 15.9 days, respectively. One (6%) patient was discharged home, and all others were discharged to an inpatient rehabilitation facility. At 6 months after surgery, all patients were alive. Ten (56%) patients had a record of follow-up 6 months after discharge. The median (IQR) time to the last documented follow-up encounter was 11.3 months (60:69.4). For comparison, a summary of postoperative complications, lengths of stay, discharge destinations and follow-up for nonagenarians undergoing TAVR are summarised in Table 7.

## Discussion

### Key Findings

In this multicentre retrospective contemporary cohort study of 18 nonagenarian patients undergoing cardiac surgery requiring cardiopulmonary bypass, we tested the primary hypothesis that mortality at 6 months would be <10% and the secondary hypothesis that serious complications would occur in >30% of patients. In keeping with our hypothesis, and despite a high median preoperative EuroSCOREs, most complications were mild to moderate, and the in-hospital and six-month mortality was zero. No patients developed organ failure, and no cerebrovascular events were recorded. Finally, while there was a loss of long-term follow-up data after 6 months, for those followed up, all were still alive at 11 months after surgery.

### Relationship to the Literature

**Figure 1:**
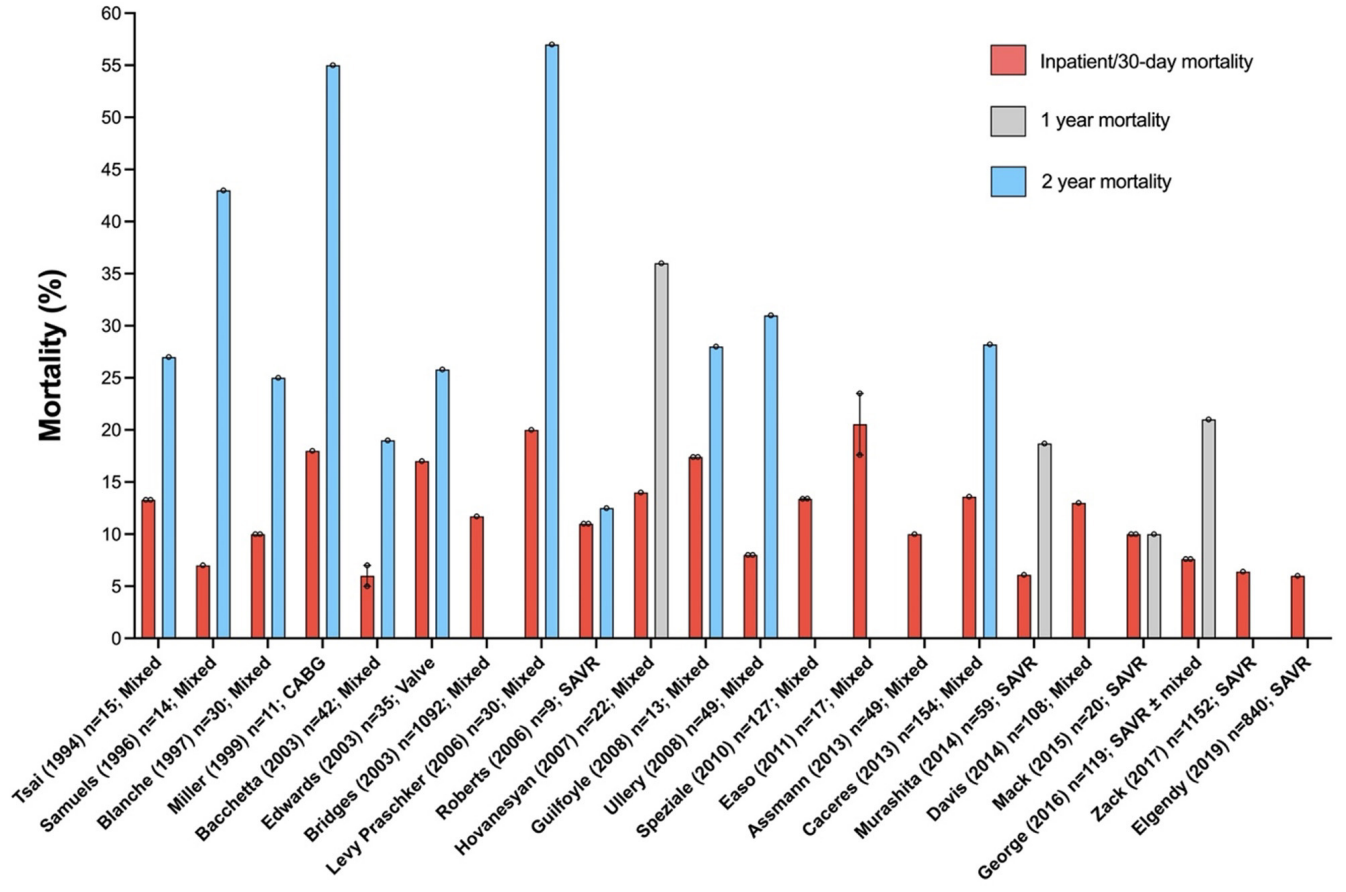
Mortality in nonagenarians undergoing cardiac surgery. Excluded: single case reports, surgery on the thoracic aorta and reports where data could not be separated by age. Error bars represent mean ± standard error of the mean.

Granular information about polypharmacy, anaesthesia medications, use of fluids, vasoactive drugs and inotropes and detailed description and grading of complications in nonagenarian patients undergoing cardiac surgery is lacking. In total, 21 studies have reported on postoperative outcomes of nonagenarians undergoing cardiac surgery (see Figure 1) (21–42). Most of these studies included <50 patients. In contrast to our outcomes, in-hospital or 30-day mortality was between 6 and 23%. Only two studies were published in the last 2 years, (28, 36) and only reported outcomes in nonagenarians undergoing surgical aortic valve replacement. Both these series, with a combined total of 1,992 patients, interrogated the same database (National Inpatient Sample database, United States) over overlapping time periods. Inpatient mortality was 6% and longer-term outcomes were not reported.

Interestingly in our series, only two patients with severe symptomatic aortic stenosis underwent isolated SAVR. This precludes direct comparisons with nonagenarians who underwent TAVR patients. In both nonagenarians who underwent SAVR, aortic stenosis was critical with severe symptoms. In both patients transfemoral access and valve and aortic morphology were unfavourable for TAVR. Over the study period our practices in the selection of nonagenarian patients for TAVR were informed by several randomized clinical trials (RCTs) comparing SAVR and TAVR, as well as large-scale registry data (43–47). Our indications for TAVR also expanded to include lower risk populations (45–50).

We favour TAVR in patients that are at increased surgical risk i.e., Society of Thoracic Surgeons (STS) or EuroSCORE II ≥4%, or other risk factors not included in these scores, such as severe frailty, porcelain aorta, sequelae of chest radiation, and presence of intact coronary artery bypass grafts when sternotomy is performed. We also favour TAVR in patients >75 years suitable for transfemoral access, unless there are anatomical or technical aspects that preclude the procedure i.e., unfavourable vascular access, short distance between the coronary ostia and aortic valve annulus, size of aortic annulus out of range for TAVR, and preclusive valve morphology (degree of calcification or calcification pattern unsuitable for TAVR). In patients with cardiac conditions that require consideration for concomitant surgical intervention (~50% of patients in our cohort), or patients at low surgical risk (STS or EuroSCORE II <4% *and* no other risk factors not included in these scores, as stated above), SAVR remains our preferred option.

Over our study period patient selection was also informed by three other clinical trials comparing TAVR and SAVR. In the PARTNER-II, SURTAVI and NOTION trials, (46–48) robust randomised data demonstrated the equivalence, and even net superiority of TAVR in intermediate-risk and lower-risk patients. Therefore, in our institution, there was a further expansion of TAVR indications to encompass lower-risk patients, as outlined in the updated 2017 ESC/EACTS guidelines on the management of valvular heart disease (51). Finally, our practices are changing given the most recent evidence that among patients aged 70 years or older with severe, symptomatic aortic stenosis and moderately increased operative risk, TAVR was non-inferior to surgery with respect to all-cause mortality at 1 year (52).

### Implications of Study Findings

Our findings suggest that with careful patient selection, comprehensive multidisciplinary care, and early involvement of geriatricians and perioperative physicians, reasonable outcomes can be achieved with acceptable morbidity and mortality in nonagenarians undergoing open cardiac surgery. Our findings also suggest that AKI, atrial fibrillation or flutter, and delirium are common in this vulnerable surgical cohort, and are targets for the implementation of specific preventative strategies. Furthermore, our study implies that the incidence of major complications was relatively low. Finally, the available data on 11-month survival supports the view that in selected nonagenarians, cardiac surgery poses an acceptable long-term mortality risk.

### Strengths and Limitations

There are several strengths to the present study. We provide a detailed description of nonagenarians who have undergone open cardiac surgery, including the granular data for key preoperative variables (including polypharmacy), detailed intra-operative variables and comprehensive postoperative outcome and complication data. The EuroSCORE-II database only contains 21 nonagenarian patients, therefore limiting its applicability to this cohort. Our results are applicable to a range of common cardiac surgeries. Data were drawn from multiple centres, using a standardised approach to define and grade complications. Finally, detailed follow-up data was available for all patients at 6 months postoperatively.

We acknowledge several limitations. First, given the retrospective study design, long-term follow-up was not available for eight patients. The small sample size together with the absence of long-term outcomes in some patients reduce the study's ability to provide a more detailed understanding of long-term outcomes. Second, the nonagenarian patients in this study were all in the lower half of the age bracket, with a maximum age of 94. As such, the findings should be applied cautiously to those of more advanced age (i.e., > 95 years of age). Third, the preponderance of men in the study may limit the applicability of its findings to women. Fourth, in defining complications as a “deviation from the normal postoperative course,” physiological aberrations expected to some degree following cardiac surgery, such as vasoplegia, were not coded as complications. Instead, these aberrations were captured by the duration and extent of the interventions used to manage them (e.g., vasopressor and ventilatory support). Similarly, certain conditions were only counted as a complication when managed with a specific intervention (e.g., pleural effusion requiring drainage). This inevitably reduced the number of complications recorded and partly predetermined their grading using the Clavien–Dindo system. Similarly, anemia in the postoperative period was also not counted as a complication given the high rate of anaemia preoperatively, with the transfusion of red blood cells instead used to provide an indication of the degree of blood loss and hemodilution.

Finally, because of the retrospective design, we acknowledge that functional and patient-centred outcomes, which are arguably more important than objective measures of morbidity and mortality in this nonagenarian cohort, could not be collected. Future studies may be enhanced by assessing functional outcome measures against metrics such as the World Health Organization's Disability Assessment Schedule (53). Further studies with larger nonagenarian sample populations and prospective study designs may also facilitate a more robust multivariable analysis. Despite these limitations, our study provides a unique description of an uncommon presentation and serves as a contemporary hypothesis generating platform for future studies.

## Conclusion

Nonagenarian patients undergoing cardiac surgery have an increased burden of comorbidity and often present for urgent or emergent surgery. Multidisciplinary patient selection and peri-operative care can lead to acceptable outcomes in nonagenarians undergoing cardiac surgery *via* median sternotomy. Further prospective studies are needed to identify risk factors associated with adverse outcomes in nonagenarians, to explore potential prophylactic measures to reduce the risk of postoperative delirium, arrhythmia complications and acute kidney injury, and to assess quality of life and functional outcomes in this vulnerable population.

## Data Availability Statement

The raw data supporting the conclusions of this article will be made available by the authors, without undue reservation.

## Ethics Statement

The studies involving human participants were reviewed and approved by Austin Health Human Research Ethics Committee. Written informed consent for participation was not required for this study in accordance with the national legislation and the institutional requirements.

## Author Contributions

LW: study conception and design, data analysis and interpretation, and writing of manuscript. DW and DL: screening of literature, patient follow up, collection of data, data analysis, and writing of manuscript. JC, LM, and BC: screening of literature, collection of data, patient follow up, and writing of manuscript. AW and TN: collection of data, follow up of patients, and writing of manuscript. SS, GM, ZA, RB, and MY: data interpretation and writing of manuscript. All authors have read and approved the manuscript.

## Conflict of Interest

The authors declare that the research was conducted in the absence of any commercial or financial relationships that could be construed as a potential conflict of interest.

## Publisher's Note

All claims expressed in this article are solely those of the authors and do not necessarily represent those of their affiliated organizations, or those of the publisher, the editors and the reviewers. Any product that may be evaluated in this article, or claim that may be made by its manufacturer, is not guaranteed or endorsed by the publisher.
